# Ticks on the Run: A Mathematical Model of Crimean-Congo Haemorrhagic Fever (CCHF)—Key Factors for Transmission

**DOI:** 10.3390/epidemiologia3010010

**Published:** 2022-03-11

**Authors:** Suman Bhowmick, Khushal Khan Kasi, Jörn Gethmann, Susanne Fischer, Franz J. Conraths, Igor M. Sokolov, Hartmut H. K. Lentz

**Affiliations:** 1 Friedrich-Loeffler-Institut, Federal Research Institute for Animal Health, Institute of Epidemiology, 17493 Greifswald, Germany; kkasi444@yahoo.com (K.K.K.); joern.gethmann@fli.de (J.G.); franz.conraths@fli.de (F.J.C.); hartmut.lentz@fli.de (H.H.K.L.); 2 Institute for Physics, Humboldt-University of Berlin, Newtonstraße 15, 12489 Berlin, Germany; sokolov@physik.hu-berlin.de; 3 Friedrich-Loeffler-Institut, Federal Research Institute for Animal Health, Institute of Infectology, 17493 Greifswald, Germany; susanne.fischer@fli.de; 4 Integrative Research Institute for the Sciences (IRIS) Adlershof, Zum Großen Windkanal 6, 12489 Berlin, Germany

**Keywords:** CCHFV, ODE, tick borne disease, targeted control, *Hyalomma*, sensitivity analysis

## Abstract

Crimean-Congo haemorrhagic fever (CCHF) is a zoonotic disease caused by the Crimean-Congo hemorrhagic fever virus (CCHFV). Ticks of the genus *Hyalomma* are the main vectors and represent a reservoir for the virus. CCHF is maintained in nature in an endemic vertebrate-tick-vertebrate cycle. The disease is prevalent in wide geographical areas including Asia, Africa, South-Eastern Europe and the Middle East. It is of great importance for the public health given its occasionally high case/fatality ratio of CCHFV in humans. Climate change and the detection of possible CCHFV vectors in Central Europe suggest that the establishment of the transmission in Central Europe may be possible in future. We have developed a compartment-based nonlinear Ordinary Differential Equation (ODE) system to model the disease transmission cycle including blood sucking ticks, livestock and human. Sensitivity analysis of the basic reproduction number $R_{0}$ shows that decreasing the tick survival time is an efficient method to control the disease. The model supports us in understanding the influence of different model parameters on the spread of CCHFV. Tick-to-tick transmission through co-feeding and the CCHFV circulation through transstadial and transovarial transmission are important factors to sustain the disease cycle. The proposed model dynamics are calibrated through an empirical multi-country analysis and multidimensional plot reveals that the disease-parameter sets of different countries burdened with CCHF are different. This information may help decision makers to select efficient control strategies.

## 1. Introduction

Crimean-Congo haemorrhagic fever (CCHF) is a tick-borne viral zoonotic disease widely distributed in Asia, Africa, Southeast Europe and the Middle East [1,2]. CCHF was first identified in an outbreak during World War II on the Crimean Peninsula in 1944–1945 [3]. The virus is antigenically identical to a virus that was isolated from the blood of a patient in Democratic Republic of the Congo in 1956. The association of these two places resulted in the name of the disease and the virus [4,5].

The etiological agent responsible for the disease, i.e., Crimean-Congo hemorrhagic fever virus (CCHFV), belongs to the genus *Orthonairovirus* in the family *Nairoviridae* [6]. CCHFV is transmitted between vertebrates and ticks but can also be transmitted horizontally and vertically within the tick population [7,8]. CCHFV persists in the tick for its whole lifespan [2]. Transmission between vertebrates and ticks occurs during blood meals [8]. Depending on the developmental stage of the ticks, i.e., larvae, nymphs and adults, the vertebrate hosts range from small (birds, hares, rabbits) to large vertebrates (cattle, sheep, humans). Animals act as viral amplifying hosts with transient viremia, but do not develop clinical signs [2]. Within the tick population, CCHFV is transstadially transmitted, venereal transmission among ticks and transmission through co-feeding may also occur [9,10]. Infected adult female ticks may lay infected eggs resulting in CCHFV-infected offspring [8]. The route of transmission through which an infected tick transfer tick-borne pathogens to a susceptible host and vice versa, is described as systemic transmission, while transmission through co-feeding, is termed non-systemic transmission.

Hard ticks of the genus *Hyalomma* are considered the main reservoir and vector for CCHFV [7,9,10]. CCHFV has also been detected in other tick genera, including *Rhipicephalus, Amblyomma, Ixodes* and *Dermacentor*, but their role in CCHFV maintenance and their vector capacity is not yet clear [2].

CCHFV is transmitted to people either by tick bites, contact with blood of infected animals or humans, body fluids or tissues. Because of severe illness and a high case fatality rate in humans, CCHF is considered as an important vector-borne disease in humans [11]. CCHF causes sporadic cases or outbreaks of severe scale across a huge geographical area extending from China to the Middle East, South-eastern Europe and Africa [1,12]. It is a highly infectious disease in humans with a case/fatality ratio of 5–80% [13]. Human cases are seasonal and associated with increased population of *H. marginatum* depending on weather conditions, habitat fragmentation and during the time of Eid-ul-Azha [14,15].

According to [16], the antibody positivity to CCHFV in livestock correlates with the manifestation of human cases and the occurrence of CCHF can happen due to contact with the blood of infected animals, e.g., in persons handling or slaughtering livestock. The review in [17] depicts the role of livestock in the maintenance and transmission of CCHFV. According to [18], high prevalence estimates in ruminants in Turkey point out the role of these animals in identifying high-risk areas for CCHF in humans. According to a sensitivity analysis of $R_{0}$ (basic reproduction number) performed in [19], the input of total host density acquires 28% of the total variability and the contribution of hare density is 16% and for the same for the cattle population is 12%.

CCHFV can spread over long distances through transportation of vectors attached to migratory birds that travel across endemic areas such as Turkey or Greece [20,21], or through imported livestock [22]. It has been estimated that every year hundreds of thousands of immature *Hyalomma* ticks are transported via migratory birds into or over Central Europe during the spring migration of birds from southern Europe and Africa [23]. The authors in [24] have developed a model to estimate the potential spread of *Hyalomma marginatum* by migratory birds into Europe. The virus has a wide range of hosts and vectors and therefore the potential to establish in a new region, if enough susceptible hosts and vectors are available. There are several factors like climate change, social and anthropogenic factors that may contribute both to the spread of CCHFV into new regions and an increase of reported cases [25]. Since several years ago, adult stages *Hyalomma marginatum* ticks have occasionally been found in Germany [23,26]. They may have been introduced by birds as nymphs and continued to develop to the adult stage [23]. In September 2018, successful moulting of a *Hyalomma* nymph removed from a horse in Dorset, England, was reported. This horse had no history of overseas travel [27]. The environmental suitability of CCHFV across Southern and Central Europe has been postulated [28]. Therefore, a rigorous yet simple and modular analysis is required to understand the potential spread and establishment of CCHFV.

Mathematical modelling can provide a tool to test different scenarios and to analyse the factors influencing the spread of CCHF. While the number of mathematical models for vector-borne diseases has generally increased, the number of mathematical models for CCHFV in particular is still limited [19,29,30,31]. We should also note that humans are an integral part of the transmission cycle and they play a major role in detecting the disease due to the frequently severe clinical course of CCHF and the high case fatality ratio in humans. So, we incorporate human in our transmission models to explore the dynamics of CCHF dissemination while accounting on sundry epidemiology. Our current modelling effort aims to provide the answers to the following questions:

1. What are the driving factors in CCHF transmission?
2. What critical density of ticks is necessary for a potential outbreak?
3. How to characterise the nature of CCHFV dissemination in the endemic areas?

We construct a deterministic Ordinary Differential Equation (ODE)-based mathematical model to investigate the fundamental mechanism of CCHFV transmission amongst the ticks, livestock, and human. In order to answer the above-mentioned questions, we have included different transmission routes of CCHFV.

## 2. Model Formulations in Different Geographic Distributions

CCHF is endemic in many regions that include Africa, Asia, Eastern and Southern Europe, Middle East but the course of transmission is not homogenous around these places [32]. Given the heterogeneity of socio-economic conditions and cultural divergences amongst these zones, potential reasons of sporadic cases of CCHF can not be explained by a single mechanism [33]. Therefore, we strive to construct reasonably simple mathematical models to determine current knowledge gaps blocking the effective control measures while taking account of different transmission routes.

**Figure 1:**
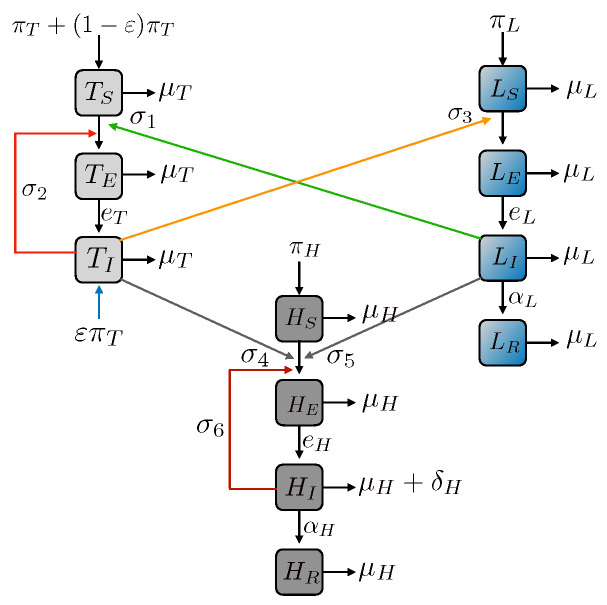
Infection process among the effective tick population (T), livestock (L), and humans (H). The infection process involves non-systemic transmission through co-feeding (red arrow), acquisition (green arrow), systemic transmission (orange arrow), influx of infected tick through transovarial transmission (blue arrow), demographic flux of individuals with different health status (black arrows) and CCHFV transmission to humans from the ticks and livestock (grey arrow) with the inclusion of human-to-human transmission (maroon arrow).

In this section, we first describe the modelling assumptions and introduce the important model parameters with their meanings. We design and analyse our compartment-based ODE model consisting of three interacting populations, i.e., ticks, livestock and humans (Figure 1) while taking account of different routes of infection. We integrate the causes of infection as tick-to-tick, tick-to-livestock, livestock-to-tick, tick-to-human, livestock-to-human and human-to-human according to the epidemiology of CCHF in different regions. The simplification of our model may help to provide the necessary general results and concurrently avoids hyper-parameterisation, while taking account of transstadial (transstadial transmission can be described as a persistent passage of a pathogen acquired during one life stage, through the moult to the next phase(s) of the vector life cycle) and transovarial transmissions (transovarial transmission can be described as the transmission of a pathogen from parent to offspring through infection of the developing egg which finally produces in infectious larva). The coupled infection-population model presented in Figure 1, concisely depicts the following mechanisms: horizontal transmission (from tick population to host) and acquisition (from livestock to tick population) of CCHFV, transstadial persistence of CCHFV throughout all phases of the tick life, transovarial transmission from infected tick females to the eggs they lay, they produce, and CCHFV transmission from infected livestock to humans along with the net growth of the interacting populations. In the diagram we also have included human-to-human transmission that we explore in Section 2.1. We opt for keeping the model as simple as possible to provide general epidemiological metrics and analytical results and, at the same including different routes of infections into the model. Then, following [34], we add together all tick stages to obtain equations for the total tick dynamics and hereby assume an effective tick population for the potential spread of CCHFV infection. This way, we avoid to model details of the infection dynamics for which there are no parameters available, such as questing rate, feeding rate, drop-off rate, and maturing parameters.

We assume that there is constant ticks’ transition rate from one developmental stage to another and that the developmental stages of the vectors function as a delay in the potential infection spread following [35]. Our main goal is to construct a mathematical model that is simple, yet including the different tick activity phases like questing or, feeding on different hosts etc., and different transmission mechanisms active. This motivates us to add together different tick phases to obtain the equations for total tick dynamics and to deduce general analytical results. We would like to make sure that our model is flexible enough to include further improvements. Thus, the resulting model includes biological mechanisms, like transovarial and transstadial transmission. Furthermore, we accommodate the feeding preferences of larvae and nymphs to include the transmittal of CCHFV burden occurring during the transstadial transmission of the respective stages. We also assume that systemic infection occurs at the beginning of the blood meal following [34]. To include the systemic and non-systemic infection, we follow [19,31,34].

We also assume that (i) Livestock has more contact with adult ticks than with other life-stages, (ii) Infection of *Hyalomma* ticks with CCHFV does not affect the birth or death rates of these ticks, (iii) Livestock will not die of CCHFV infection [2], while CCHF-induced deaths in humans are taken into account [13].

So, we construct an ODE based model including the above-mentioned assumptions of CCHFV transmission to analyse the burden of primary transmission routes and attribute the predominant role of CCHFV dissemination to the effective tick population to simplify the modelling effort. For the effective tick population, we use an SEI dynamics, as the tick remains infected for life [2], while for the livestock and human populations we assume SEIR type of dynamics. We include the exposed state in the models as the ticks become infected after a blood meal while maturing to the next phases of developmental stages [36].

Considering the aforementioned assumptions, we establish the following model system: For the tick population we have

(1) $$\begin{array}{ccc} \frac{dT_{S}}{dt} & = & \pi_{T}-\frac{\sigma_{1}T_{S}L_{I}}{L}-\frac{\sigma_{2}T_{S}T_{I}}{T}-\mu_{T}T_{S}+\left(1-\varepsilon \right)\pi_{T}T_{I}\\ \frac{dT_{E}}{dt} & = & \frac{\sigma_{1}T_{S}L_{I}}{L}+\frac{\sigma_{2}T_{S}T_{I}}{T}-\mu_{T}T_{E}-e_{T}T_{E}\\ \frac{dT_{I}}{dt} & = & e_{T}T_{E}-\mu_{T}T_{I}+\varepsilon \pi_{T}T_{I}. \end{array}$$

Following [35], we assume that the tick demography is density-dependent. In order to take the density dependence into account and simplifying further, we consider a negative exponential model for the tick demography, i.e., higher abundance of ticks reduces their reproduction. Therefore, we model the reproduction rate $\pi_{T}=\sigma_{T}\omega_{T}\exp\left(-\nu_{T}\frac{T_{S}+T_{E}+T_{I}}{\varrho \omega_{1}+L\omega_{2}}\right)$ following [19,35], where $T_{S}$, $T_{E}$, $T_{I}$ are susceptible, exposed and infected ticks respectively, $\nu_{T}$ is the strength of density-dependence in hatching rate, $\omega_{1}$ and $\omega_{2}$ are the weightage of contributions of rodents or the small mammals and livestock populations on the growth of ticks, respectively, $\sigma_{T}$ is detachment rate of tick and $\omega_{T}$ is the mean number of eggs laid by an adult female tick, ϱ is the constant number of rodents or the small mammals and *L* is the total number of livestock. We incorporate the rodents or the small mammals-tick transmission cycle without explicitly deriving the equations of different stages of ticks and rodent population, as analytical computations become inconvinient and the model would become too complex. We include transovarial transmission from adult female ticks to eggs after introducing a parameter ε that measures the proportion of infected eggs laid by an infected female adult tick as mentioned in [35]. We have also opted for a density-dependent mortality rate ($\mu_{T}$) according to [19] and it is defined as $\mu_{T}=\mu_{0}+\alpha_{0}\ln\left(1+\frac{T_{i}}{L}\right)$, where *i* stands for S, E, I, $\mu_{0}$ and $\alpha_{0}$, respectively defines the basal rate and the factor that defines the influence of host density.

We now explain the formulations of transmission parameters of the tick related model system. Livestock-to-tick infection transmission rate has been modelled according to [8,31] as $\sigma_{1}=\frac{p_{T}\gamma_{T}N_{T}f_{S}}{d_{T}}$, where $p_{T}$ is defined as the transmission efficiency from livestock to tick, $\gamma_{T}$ is the duration of infective period, $N_{T}$ is the rate of average number of feeding ticks on livestock, $f_{S}$ is defined as the fraction of blood meal per tick and $d_{T}$ is duration of attachment. Once an infected nymph (for example) feeds on a host, it will infect a certain fraction of the other ticks feeding on the same host during the entire blood meal time (transmission by co-feeding). According to [35,37], transmission by co-feeding depends on the encounter rate, mean or the total number of other ticks and the probability of pathogen transmission.

Non-systemic transmission term $\sigma_{2}$ is defined according to [34] as follows: $\sigma_{2}=\left[1-\exp\left\{-(n_{\varrho }+l_{\varrho })\theta \right\}\right]\sigma_{\varrho }\varrho$, where $n_{\varrho }$ is the fraction of nymphs against the total number of ticks feeding on rodents, $l_{\varrho }$ is the fraction of larvae against the total number of ticks feeding on rodents, θ is the transmission probability, $\sigma_{\varrho }$ is an encounter rate between ticks and rodents.

The domestic livestock population is described by the following system of equations:

(2) $$\begin{array}{ccc} \frac{dL_{S}}{dt} & = & \pi_{L}-\frac{\sigma_{3}L_{S}T_{I}}{T}-\mu_{L}L_{S}\\ \frac{dL_{E}}{dt} & = & \frac{\sigma_{3}L_{S}T_{I}}{T}-e_{L}L_{E}-\mu_{L}L_{E}\\ \frac{dL_{I}}{dt} & = & e_{L}L_{E}-\alpha_{L}L_{I}-\mu_{L}L_{I}\\ \frac{dL_{R}}{dt} & = & \alpha_{L}L_{I}-\mu_{L}L_{R}. \end{array}$$

The human population is described by the following system of equations:

(3) $$\begin{array}{ccc} \frac{dH_{S}}{dt} & = & \pi_{H}-\frac{\sigma_{4}H_{S}T_{I}}{T}-\frac{\sigma_{5}H_{S}L_{I}}{L}-\mu_{H}H_{S}\\ \frac{dH_{E}}{dt} & = & \frac{\sigma_{4}H_{S}T_{I}}{T}+\frac{\sigma_{5}H_{S}L_{I}}{L}-e_{H}H_{E}-\mu_{H}H_{E}\\ \frac{dH_{I}}{dt} & = & e_{H}H_{E}-\alpha_{H}H_{I}-\mu_{H}H_{I}-\delta_{H}H_{I}\\ \frac{dH_{R}}{dt} & = & \alpha_{H}H_{I}-\mu_{H}H_{R}. \end{array}$$

Here, $L_{S}$, $L_{E}$, $L_{I}$, $L_{R}$ and L represent susceptible, exposed, infected, recovered and total livestock, respectively, and similarly $H_{S}$, $H_{E}$, $H_{I}$, $H_{R}$ and H do the same for the human population.

We also assume a density-dependent birth rate ($\pi_{L}$) for livestock and a linear growth rate ($\pi_{H}$) for humans. The mortality rates are not density depended.

After blood feeding, ticks start to develop to the next stage in their life cycle and thereby the pathogen can propagate between these stages. The duration of the development is assumed to be negative exponentially distributed, as mentioned before. Between the stages there are different mean values for the durations [35,38].

We model the acquisition rate as $\sigma_{3}=\left[1-\exp\left\{-(N_{\varrho }\kappa_{N}+L_{\varrho }\kappa_{L}+A_{L}\kappa_{A})\right\}\right]\sigma_{L}L$ after [19,31] to include the propagation of infection acquired by the transstadial stages in a simplified way. Here $\kappa_{i}$ is the transmission rate from larvae, nymphs and adult ticks, where i=L,N,A. $N_{\varrho }$ is the ratio between the infectious nymphs and constant rodent density, $L_{\varrho }$ is the ratio between the infectious larvae and constant rodent density, $A_{L}$ is the ratio between the infectious adult ticks and livestock density and $\kappa_{i}=1-{\left(1-T_{i}\right)}^{\frac{1}{d_{feed_{i}}}}$, with $T_{i}$ is the overall efficiency of transmission, $d_{feed_{i}}$ is the dimensionless feeding duration (measured in number of time-steps) and $\sigma_{L}$ is encounter rate between the ticks and livestock. $\sigma_{4}$ is the transmission rate from an infected tick to a susceptible human and $\sigma_{5}$ is the transmission rate from an infected livestock to a susceptible human. A full list of model parameters, variables and their biological meanings are provided in Table 1 and in the Supplementary Information (SI) (Tables S1–S3).

### 2.1. Inclusion of Human-to-Human Transmission

CCHF is a viral zoonosis with cases of human-to-human transmission [45,46,47]. To take account of human-to-human transmission ($\sigma_{6}$), we include the respective route [48] in the model as depicted in Figure 1. The description of $\sigma_{6}$ is included in the Supplementary Information (SI) (Equation (S5)).

Thus, our model equation system described in (3) modifies into the following:

(4) $$\begin{array}{ccc} \frac{dH_{S}}{dt} & = & \pi_{H}-\frac{\sigma_{4}H_{S}T_{I}}{T}-\frac{\sigma_{5}H_{S}L_{I}}{L}-\frac{\sigma_{6}H_{S}H_{I}}{H}-\mu_{H}H_{S}\\ \frac{dH_{E}}{dt} & = & \frac{\sigma_{4}H_{S}T_{I}}{T}+\frac{\sigma_{5}H_{S}L_{I}}{L}+\frac{\sigma_{6}H_{S}H_{I}}{H}-e_{H}H_{E}-\mu_{H}H_{E}\\ \frac{dH_{I}}{dt} & = & e_{H}H_{E}-\alpha_{H}H_{I}-\mu_{H}H_{I}-\delta_{H}H_{I}\\ \frac{dH_{R}}{dt} & = & \alpha_{H}H_{I}-\mu_{H}H_{R}. \end{array}$$

### 2.2. Tick-Human Model

According to [16,49], many of the reported cases of CCHFV are due to bites by adult ticks. As reported in [16], *Hyalomma* are “hunting” ticks and they can chase up to 400 m to find their hosts (including humans). Due to occupational exposure to the bites by infected ticks or crushing infected ticks with bare hands, humans can be infected, too [50]. The study in [51] reported a large number of patients tested positive for CCHFV due to potential exposure via tick bites along with asymptomatic cases of CCHF in Tajikistan. A survey conducted in Turkey revealed that among all reported cases, 68.9% had a history of tick-bites or contact with ticks, while 0.16% cases represented nosocomial infections [52]. The latter represents transmission during the process of receiving health care, when humans can get in contact with infected tissue.

In order to model the CCHFV transmission during slaughtering and meat handling under ideal hygiene conditions and to take into account the above mentioned tick-human transmission of CCHFV, we consider only the subsystem related to human and tick while ignoring the livestock-to-human transmission path, (1), (2), (4). Consequently, the resulting reduced model can be treated like a classic vector-host model while simplifying the underneath complicated biology. The deriving mathematical model can be employed to deduce the epidemiological threshold conditions and that can potentially be helpful for the effective control of CCHF. Consequently, after following the authors in [29], we leave out the livestock-to-tick by assuming $\sigma_{1}=0$. We obtain the following ODE system:

(5) $$\begin{array}{ccc} \frac{dT_{S}}{dt} & = & \pi_{T}-\frac{\sigma_{2}T_{S}T_{I}}{T}-\mu_{T}T_{S}+\left(1-\varepsilon \right)\pi_{T}T_{I}\\ \frac{dT_{E}}{dt} & = & \frac{\sigma_{2}T_{S}T_{I}}{T}-\mu_{T}T_{E}-e_{T}T_{E}\\ \frac{dT_{I}}{dt} & = & e_{T}T_{E}-\mu_{T}T_{I}+\varepsilon \pi_{T}T_{I}. \end{array}$$

(6) $$\begin{array}{ccc} \frac{dH_{S}}{dt} & = & \pi_{H}-\frac{\sigma_{4}H_{S}T_{I}}{T}-\frac{\sigma_{6}H_{S}H_{I}}{H}-\mu_{H}H_{S}\\ \frac{dH_{E}}{dt} & = & \frac{\sigma_{4}H_{S}T_{I}}{T}+\frac{\sigma_{6}H_{S}H_{I}}{H}-e_{H}H_{E}-\mu_{H}H_{E}\\ \frac{dH_{I}}{dt} & = & e_{H}H_{E}-\alpha_{H}H_{I}-\mu_{H}H_{I}-\delta_{H}H_{I}\\ \frac{dH_{R}}{dt} & = & \alpha_{H}H_{I}-\mu_{H}H_{R}. \end{array}$$

## 3. Basic Reproduction Number $R_{0}$

The initial spread of epidemics can be described by the *basic reproduction number* ($R_{0}$). Elementarily, it characterises the expected number of secondary cases produced by a single primary case in a completely susceptible population. To illustrate the spread of pathogens that infect multiple hosts, a formal mathematical framework named *Next Generation Matrix* (NGM) has been developed in [53]. The elements of the NGM ($K_{ij}$) are the expected number of infected individuals of type *i* produced by a single infectious individual of type *j*. We address the question, under which conditions the virus can spread in a completely susceptible population, given a single infected individual is introduced. Mathematically, we analyse the stability of the disease-free equilibrium $E_{0}$, which is a fixed point of the system (1)–(4). It is given by $E_{0}=\left(T_{S}^{*},0,0,L_{S}^{*},0,0,0,H_{S}^{*},0,0,0\right)=\left(\frac{\pi_{T}^{0}}{T},0,0,\frac{\pi_{L}^{0}}{L},0,0,0,\frac{\pi_{H}^{0}}{H},0,0,0\right)$. Information regarding the mathematical properties of the model solutions and a stability analysis are provided in the Supplementary Information (SI) (Sections S1 and S4). If $E_{0}$ is stable, the disease dies out before it can infect individuals, and it can spread over the population, if $E_{0}$ is unstable. The stability condition for $E_{0}$ can be expressed in terms of $R_{0}$, where the outbreak condition is $R_{0}>1$. To compute the basic reproduction number, we use the NGM method as described in [54]. Detailed computation is included in the Supplementary Information (SI) (Section S3). Next, we derive a mathematical expression of $R_{0}$ under different assumptions considered in the Section 2, Section 2.1 and Section 2.2.

The NGM associated with the model (1)–(3) of Section 2 can be written as follows:

(7) $$K_{\mathrm{TL}}=\left(\begin{array}{cc} \frac{T_{S}^{*}e_{T}\sigma_{2}}{T\left(e_{T}+\mu_{T}\right)\left(\mu_{T}-\varepsilon \pi_{T}^{0}\right)} & \frac{T_{S}^{*}e_{L}\sigma_{1}}{L\left(\alpha_{L}+\mu_{L}\right)\left(e_{L}+\mu_{L}\right)}\\ \frac{L_{S}^{*}e_{T}\sigma_{3}}{T\left(e_{T}+\mu_{T}\right)\left(\mu_{T}-\varepsilon \pi_{T}^{0}\right)} & 0 \end{array}\right).$$

It can be biologically interpreted as

(8) $$K_{\mathrm{TL}}=\left(\begin{array}{cc} Tick↪Tick & Livestock↪Tick\\ Tick↪Livestock & 0 \end{array}\right),$$

where X↪Y means population *X* is infecting population *Y*. It should be noted that Tick↪Tick in this context means transmission within the whole tick population due to co-feeding as well as transovarial transmission.

The basic reproduction number is defined as the spectral radius of the NGM (7). In our model, we decompose the total basic reproduction number $R_{0}$ into different contributions. These are (i) infection from tick to tick via co-feeding and vertical transmission ($R_{T}$) and (ii) infection from tick to livestock ($R_{LA}$). For the whole model we get

(9) $$\begin{array}{ccc} R_{0} & = & \frac{R_{T}}{2}+\sqrt{{\left(\frac{R_{T}}{2}\right)}^{2}+R_{LA}}\\ & = & \frac{1}{2}\left[R_{T}+\sqrt{R_{T}^{2}+4R_{LA}}\right]. \end{array}$$

The epidemic threshold is the critical point, where $R_{0}=1$.

In Equation (9),

(10) $$R_{T}=\left[\frac{\pi_{T}^{0}}{T}\frac{e_{T}}{\left(e_{T}+\mu_{T}\right)}\frac{\sigma_{2}}{\left(\mu_{T}-\varepsilon \pi_{T}^{0}\right)}\frac{1}{\mu_{T}}\right]$$

is the contribution of transmission within the tick population due to co-feeding and transovarial transmission and

(11) $$R_{LA}=\left[\left(\frac{\pi_{T}^{0}}{T}\frac{e_{L}}{\left(e_{L}+\mu_{L}\right)}\frac{1}{\left(\alpha_{L}+\mu_{L}\right)}\frac{\sigma_{1}}{\mu_{L}}\right)\left(\frac{\pi_{L}^{0}}{L}\frac{e_{T}}{\left(e_{T}+\mu_{T}\right)}\frac{\sigma_{3}}{\left(\mu_{T}-\varepsilon \pi_{T}^{0}\right)}\frac{1}{\mu_{T}}\right)\right]$$

is the contribution of tick-to-livestock and livestock-to-tick transmission. Equation (9) can also be represented as:

(12) $$R_{0}=\frac{Tick↪Tick}{2}+\sqrt{{\left(\frac{Tick↪Tick}{2}\right)}^{2}+\left(Livestock↪Tick\right)\left(Tick↪Livestock\right)}$$

**Figure 2:**
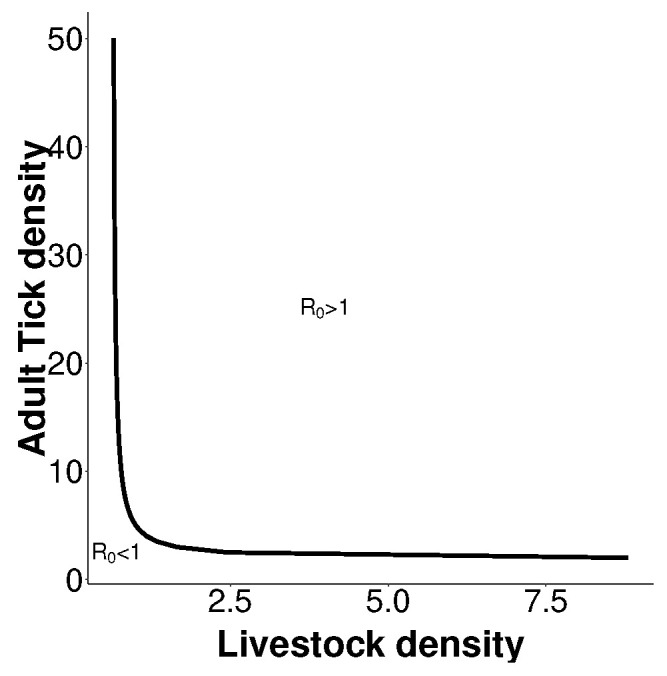
Relationship between tick density and livestock density in an area of predicted CCHF persistence. Parameters values are used from Table 1.

We explore the effect of livestock density on the number of effective tick population as derived in (9) after drawing the curve what is described by $R_{0}=1$ and observe from the Figure 2 the required density of expected livestock that will lead to the persistence of CCHF.

The curve for $R_{0}=1$ illustrates the possible expected cut-off point for CCHFV to persist, theoretically. Above this curve, CCHFV will persist, while it will die out below the curve.

If we exclude the transmission within the tick population, then the basic reproduction number is simply $R_{0}^{w}=\sqrt{R_{LA}}$. $R_{0}^{w}$ can biologically be described as $R_{0}^{w}=\sqrt{\left(Livestock↪Tick)(Tick↪Livestock\right)}$.

The terms in (10) can be interpreted as follows: $\frac{e_{T}}{e_{T}+\mu_{T}}$ is the probability that a tick will survive the incubation period and become infectious after co-feeding, $\frac{1}{\mu_{T}}$ is the natural lifespan of a tick, $\frac{\sigma_{2}}{\mu_{T}-\varepsilon \pi_{T}^{0}}$ is the probability of CCHFV transmission from a tick to another tick through non-systemic and transstadial transmission in its lifetime. $\frac{\pi_{T}^{0}}{T}$ is the ratio between the birth rate of a tick and the total number of ticks, where $\pi_{T}^{0}$ is the susceptible tick population at the disease free equilibrium $E_{0}$ as explained at the beginning of this section. In the same way, the terms in (11) can be explained as before.

$\frac{e_{L}}{e_{L}+\mu_{L}}$ is the proportion of livestock that will survive the incubation period and become infectious,

$\frac{1}{\alpha_{L}+\mu_{L}}$ is the infectious lifespan of this livestock, $\frac{\sigma_{1}}{\mu_{L}}$ is the probability of CCHFV transmission from the livestock to a tick in the lifespan of infectious livestock,

$\frac{\sigma_{3}}{\mu_{T}-\varepsilon \pi_{T}^{0}}$ is the probability of CCHFV transmission from an infectious tick to livestock during the span of its natural growth, and the total number of livestock and $\frac{\pi_{L}^{0}}{L}$ is the ratio between the birth rate of livestock and the total number of livestock. Finally, using (9) with the minimum values of the parameters provided in Table 1, we obtain the following figure for the basic reproduction number:

(13) $$R_{0}=3.4,$$

where the contributions are for co-feeding and transovarial transmission $R_{T}=1.6$, and for the tick-to-livestock and livestock-to-tick infection $R_{LA}=7.2$. When we perform the same calculations with the maximum values of the parameters, we get $R_{T}=2.4$, $R_{LA}=10.75$, and $R_{0}=4.9$, respectively.

After including human-to-human transmission [46,55,56], the model system (1), (2), (4) has a next generation matrix that can be simplified to

(14) $$K_{\mathrm{TLH}}=\left(\begin{array}{ccc} \frac{T_{S}^{*}e_{T}\sigma_{2}}{T\left(e_{T}+\mu_{T}\right)\left(\mu_{T}-\varepsilon \pi_{T}^{0}\right)} & \frac{T_{S}^{*}e_{L}\sigma_{1}}{L\left(\alpha_{L}+\mu_{L}\right)\left(e_{L}+\mu_{L}\right)} & 0\\ \frac{L_{S}^{*}e_{T}\sigma_{3}}{T\left(e_{T}+\mu_{T}\right)\left(\mu_{T}-\varepsilon \pi_{T}^{0}\right)} & 0 & 0\\ \frac{H_{S}^{*}e_{T}\sigma_{4}}{T\left(e_{T}+\mu_{T}\right)\left(\mu_{T}-\varepsilon \pi_{T}^{0}\right)} & \frac{H_{S}^{*}e_{L}\sigma_{5}}{L\left(\alpha_{L}+\mu_{L}\right)\left(e_{L}+\mu_{L}\right)} & \frac{H_{S}^{*}e_{H}\sigma_{6}}{H\left(\alpha_{H}+\delta_{H}+\mu_{H}\right)\left(e_{H}+\mu_{H}\right)} \end{array}\right)$$

with spectral radius

(15) $$R_{0}=\max\left[R_{H},R_{LA}\right]$$

where

(16) $$R_{H}=\left[\frac{\pi_{H}^{0}}{H}\frac{\sigma_{6}}{\mu_{H}}\frac{e_{H}}{\left(e_{H}+\mu_{H}\right)}\frac{1}{\left(\alpha_{H}+\delta_{H}+\mu_{H}\right)}\right]$$

The matrix $K_{\mathrm{TLH}}$ (14) can be biologically interpreted as

(17) $$K_{\mathrm{TLH}}=\left(\begin{array}{ccc} Tick↪Tick & Livestock↪Tick & 0\\ Tick↪Livestock & 0 & 0\\ Tick↪Human & Livestock↪Human & Human↪Human \end{array}\right)$$

Next generation matrix $\left(K_{\mathrm{TH}}\right)$ associated with (5), (6) is given by

(18) $$K_{\mathrm{TH}}=\left(\begin{array}{cccc} \frac{T_{S}^{*}e_{T}\sigma_{2}}{T\left(e_{T}+\mu_{T}\right)\left(\mu_{T}-\varepsilon \pi_{T}^{0}\right)} & \frac{T_{S}^{*}\sigma_{2}}{T(\mu_{T}-\varepsilon \pi_{T}^{0})} & 0 & 0\\ 0 & 0 & 0 & 0\\ \frac{H_{S}^{*}e_{T}\sigma_{4}}{T\left(e_{T}+\mu_{T}\right)\left(\mu_{T}-\varepsilon \pi_{T}^{0}\right)} & \frac{H_{S}^{*}\sigma_{4}}{T(\mu_{T}-\varepsilon \pi_{T}^{0})} & \frac{H_{S}^{*}e_{H}\sigma_{6}}{H\left(\alpha_{H}+\delta_{H}+\mu_{H}\right)\left(e_{H}+\mu_{H}\right)} & \frac{H_{S}^{*}\sigma_{6}}{H\left(\alpha_{H}+\delta_{H}+\mu_{H}\right)}\\ 0 & 0 & 0 & 0 \end{array}\right)$$

(19) $$R_{TH}=\max\left[R_{H},R_{T}\right]$$

when we consider the whole model, but exclude the livestock-to-humans transmission, we can derive the basic reproduction number of the decoupled system as $R_{TH}=\max\left[R_{H},R_{LA}\right]$. In order to understand $R_{LA}$, $R_{TH}$ and the stability of the disease free equilibrium point, we put forward a scaling factor η that can be a potential gauge to change the transmission rates to model control programs. Following [57], we replace the host-specific transmission rates ($\sigma_{1}\to \eta \sigma_{1}$, $\sigma_{3}\to \eta \sigma_{3}$, $\sigma_{4}\to \eta \sigma_{4},\sigma_{6}\to \eta \sigma_{6}$) and calculate the value of $R_{H}$ where η∈[0,1]. $R_{TH}$ becomes less than one when η≈0.37 and for $R_{LA}$, the value is η≈0.18. The influence of η is included in the Supplementary Information (SI) (Figure S2).

## 4. Dynamics of the Model

**Figure 3:**
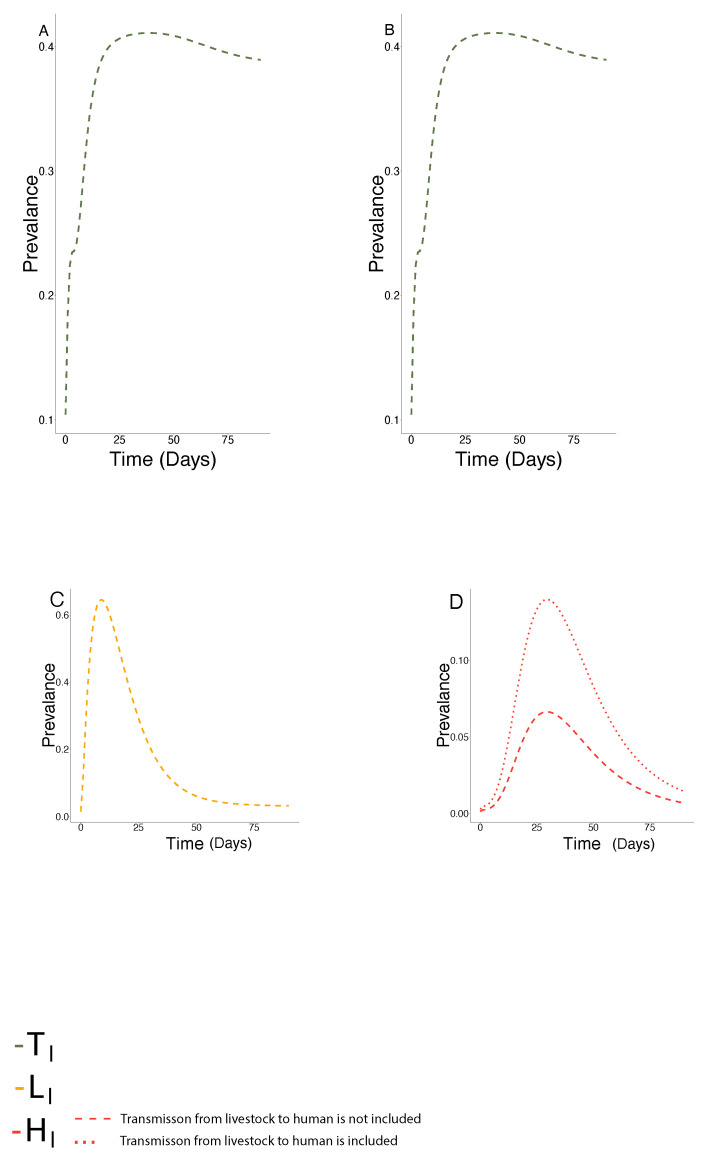
Simulated infection dynamics of models (1)–(3), (5) and (6). CCHFV prevalence in the effective tick population (**A**,**B**), livestock populations (**C**) and human (**D**) while considering in this instance both infectious and removed host populations according to [19].

In this section, we carry out simulations to study the long time dynamics of our model system (1)–(3), (5) and (6) that we have formulated using the parameters given in Table 1. Figure 3 depicts the temporal dynamics of the model system when we take account of human-to-human transmission along with the transmission routes from livestock-to-human, and without considering it. The initial conditions are described in the Supplementary Information (SI) (Table S2).

The simulation experiments reveal that the CCHFV prevalence in the ticks does not show large variations with time and it saturates at around 40%. The simulated prevalence in livestock shows the highest prevalence around 62% (bottom panel) and it decreases slowly. The simulated prevalence in humans in both cases (Figure 3D) reveals the importance of the dissemination of CCHFV from livestock-to-humans in the countries of concern. Moreover, the prevalence in humans is doubled if we include livestock to human transmission.

## 5. Control Strategies in Different Geographic Locations

We use our model to analyse different control measures that could be employed by policy makers to decrease the number of human cases and the duration of outbreak situations in a theoretical setting. With the presented multi-host model, the exploration of all possible control strategies is difficult to undertake. This leaves us with the choice of aiming at particular host types only, such as ticks, e.g., for vector control, humans, e.g., for social distancing among humans, or livestock, e.g., for vaccination, treatment with acaricides or other means to reduce tick infestation etc. An epidemiological metric named *target reproduction number* ($T_{S}$) was defined to quantify the control measurements for infectious diseases with multiple host types [58]. This metric can be applied to study various control measures, when targeting a subset of different types of hosts. Let us denote K from (14) as follows for convenience:

(20) $$K=\left(\begin{array}{ccc} K_{11} & K_{12} & 0\\ K_{21} & 0 & 0\\ K_{31} & K_{32} & K_{33} \end{array}\right)$$

There are several options, through which we can curb the bite of CCHFV-infected ticks. The general idea is to represent control strategies as a target set S being a subset of the entries of K. Keeping the same notations as in [59], the target reproduction number $T_{S}$ with respect to the target set S is defined as

(21) $$T_{S}=\rho \left(K_{S}{\left(I-K+K_{S}\right)}^{-1}\right)$$

where, $K_{S}$ is the target matrix and defined as ${\left[K_{S}\right]}_{ij}=K_{ij}$, if (i,j)∈S and 0, otherwise. I is the identity matrix and ρ is the spectral radius of the matrix (ρ should not be confused with the rodent population ϱ used above). Different disease control strategies are described below:

*Livestock Sanitation:* The usage of acaricides is a common technique to lower the tick burden in the livestock population. Then the target set is S={(1,2),(2,1),(3,2)}, representing index pairs in (20). The type reproduction number targeting the host type 1 after employing (21) is given by: $T_{S}=\sqrt{\frac{K_{12}K_{21}}{1-K_{11}}}$, provided $K_{11}<1$.

*Human Sanitation & Isolation:* The target set is S={(3,1),(3,2),(3,3)}. Target reproduction number $T_{S}$ with respect to S (i.e., the type reproduction number targeting the host type 2) is given by $T_{S}=K_{33}$.

The rest of the options is included in the Supplementary Information (SI) (Section S11).

## 6. Sensitivity Analysis

In this section we carry out a sensitivity analysis of the model parameters to the model output to deduce the important parameters that may help to understand the significant parameters responsible for the CCHF infection. It can be understood as the treatise of how uncertainty in the output of a mathematical model corresponds to different sources of uncertainty in the model input parameters [60].

### Model Sensitivity Analysis

We perform a sensitivity analysis through computing the Partial Rank Correlation Coefficients (PRCC) with 1000 simulations per run for each of the model input parameter values sampled by the Latin Hypercube Sampling (LHS) scheme to obtain those model parameters that have the greatest influence on CCHFV transmission. We consider the cumulative human cases of CCHF occurring during a simulation experiment as the model output of interest without including human-to-human spread. This approach has the advantage that it captures the effects of model parameters on both, the persistence of CCHF and the overall impact of CCHF outbreaks over time.

We have used the *sensitivity* package [61], and for the LHS scheme we have utilised the *lhs* package [62] in R [63].

**Figure 4:**
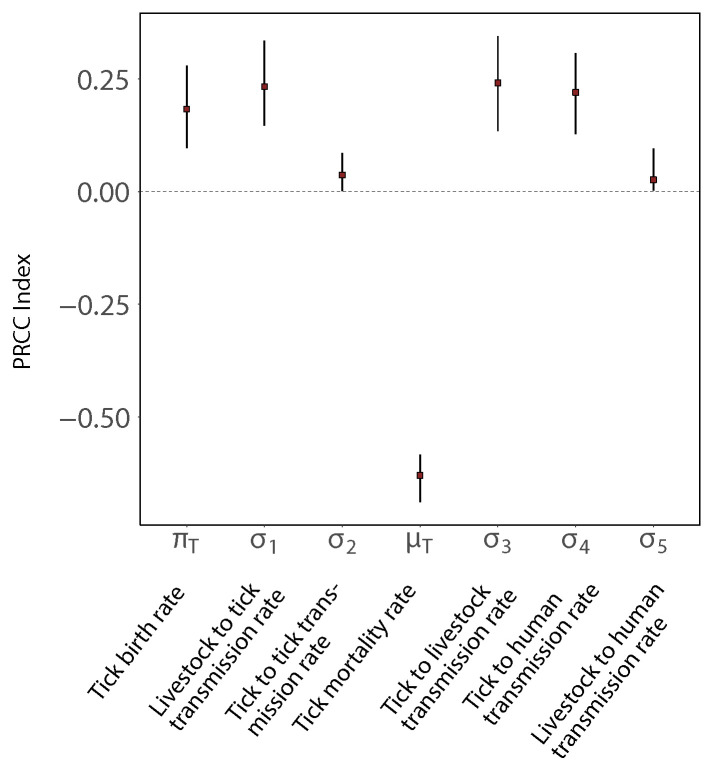
Sensitivity Analysis of the model using partial rank correlation coefficients. Only parameters with significant impact are shown. For all parameters see Supplementary Information (SI) (Section S12).

In Figure 4 we show the PRCCs for the most significant parameters. We observe that the mortality of the infected ticks ($\mu_{T}$) shows a strong negative correlation with the cumulative incidences of CCHF in humans, whereas the effective transmission between tick and livestock ($\sigma_{3}$, $\sigma_{1}$), the tick reproduction rate ($\pi_{T}$), the effective transmission between ticks and humans ($\sigma_{4}$) show strong positive correlations with the model output, whereas the effective transmission rate between livestock and humans ($\sigma_{5}$) and the effective transmission amongst the ticks through co-feeding ($\sigma_{2}$) are not so significant. Therefore, we conclude that the parameters with the strongest influence on the cumulative incidences of CCHF in humans are $\mu_{T}$, $\pi_{T}$ and $\sigma_{i}$, where (i=1,3,4). Cumulative incidences of CCHF in human increase with an increase in $\pi_{T}$ and $\sigma_{i}$, where (i=1,3,4) and decrease with $\mu_{T}$.

## 7. Fitting the Model to Outbreak Data

Even if data for most parameters of our models are available, the real prevalence of the different compartments in vectors and hosts remain unknown, and thus the model is hard to validate. We therefore calibrate our model using data on infections in humans being a proxy for the prevalence in all relevant species.

To calibrate our ODE model (5), (6), we have fitted it to the CCHFV reported incidence data from six different countries focusing on the unknown transmission parameters $\sigma_{2}$, $\sigma_{4}$, and $\sigma_{6}$. The rest of the parameters values are used from Table 1. After employing the MATLAB^®^ [64] differential equation solver *ode45*, we have used the Matlab functions *fminsearch* and *lsqcurvefit*.

**Figure 5:**
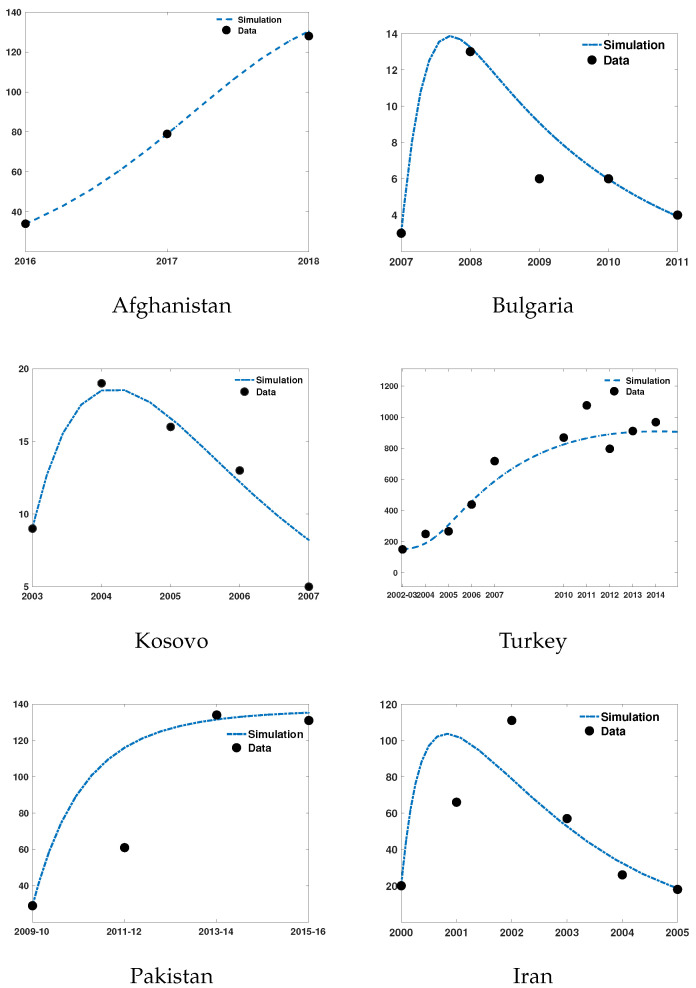
Reported human CCHFV cases in Afghanistan, Bulgaria, Kosovo, Turkey, Pakistan and Iran and the simulation of $H_{I}\left(t\right)$ from the model fitted to these data points. The outbreak data are all collected from [11,15,65,66,67,68].

The fitted numerical solutions of human CCHFV cases in different countries are shown in Figure 5. The increased awareness towards the perils of CCHFV may have helped to decrease the number of cases for Bulgaria, Iran and Kosovo, but in other countries it appears that this is not the case. Moreover, our fitted model simulations (Figure 5) demonstrate that, given the current trend of CCHFV cases in Afghanistan, Pakistan, and Turkey, the number of human CCHFV cases might keep on increasing in future. Therefore, if no further effective prevention and control measures are taken, the disease will not vanish. Values of the fitted parameters are included in the Supplementary Information (SI) (Figure S3).

### Comparison of Fitted Transmission Parameters

**Figure 6:**
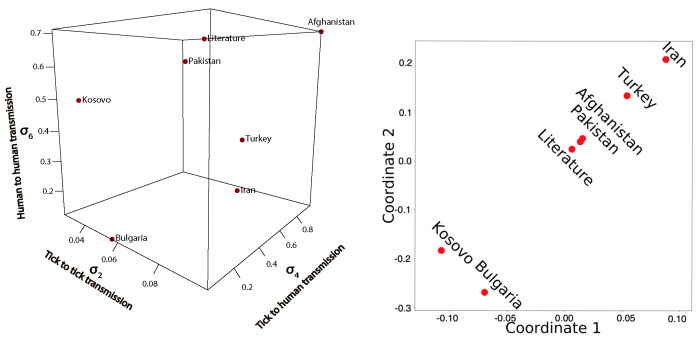
Scattered plot of the fitted transmission parameters and the values from the literatures [31,34,35,44] and the embedded multidimensional scaling plot of the fitted parameters.

A central question pertaining to the fitted parameters of different countries is: how do the parameters differ from each other? In order to answer this question, we plot the fitted transmission coefficients along with the values retrieved from the literature [31,34,35,44] in Figure 6. The data fitting is restricted to the transmission parameters $\sigma_{2}$, $\sigma_{4}$ and $\sigma_{6}$.

From Figure 6 it is evident that the disease transmission parameters differ from country to country. We can clearly observe that Afghanistan is the most affected country and has the highest disease transmission burden. The estimated transmission parameters of Afghanistan, Pakistan, Iran, Turkey and the literature values are closer to each other as compared to Bulgaria and Kosovo. We also have included multidimensional scaling (mds)(see Figure 6) to visualise the level of cosine similarity or dissimilarity of the fitted transmission parameters of concerned countries. Visualisations of the resulting heat-map is included in the Supplementary Information (SI) (Figure S4).

## 8. Discussion and Conclusions

We analyse a new mathematical model that includes multiple transmission routes of CCHFV and compare it with a model that only incorporates the transmission of the CCHFV virus through direct contact with livestock and ticks. Our model shows the importance of including CCHFV transmission through co-feeding and its sustainability in the tick population. Co-feeding is an essential biological mechanism for the tick population and increases the basic reproduction number. With the assumed parameter list, our model predicts that a reduction of 18% of the contact rate between the ticks and the livestock can reduce the value of the basic reproduction number below the epidemic threshold, which will ultimately lead to the dying out of the disease. When we consider only the tick-human model in Section 2.2, we observe that a reduction of 37% in the contact rate helps to reduce the value of the basic reproduction below 1.

Our simulations show that livestock has a significant role in disease transmission (compared to the exclusive tick-human model), especially during the time of Eid-ul-Azha. There is an increase of approximately 50% in human CCHFV prevalence due to contact with infected animal tissue. We propose that these additional pathways do not only increase the basic reproductive number of CCHF, but also have a dominant role in CCHF control and require appropriate measures.

The computation of $R_{0}$ gives us the necessary tool to investigate different strategies to control the spread of vectors-borne diseases. The results derived from the sensitivity analysis suggest the importance of birth rate of tick population in the potential spread of CCHFV transmission, and influence of rodent density as one can observe the functional dependence of the birth rate of the ticks depends on the density of the rodent population. Linear growth in the tick population is reflected in the basic reproduction number (both in $R_{LA}$ and $R_{T}$). Freshly recruited naive livestock provides the necessary number of susceptible livestock, which increases the value of the basic reproduction number. Transmission of CCHFV from tick to livestock is an important parameter and the increase in effective contact between questing adult ticks and the livestock species increases the basic reproduction number in a nonlinear way.

Some of the results are in accordance with the findings of previous studies. For example, the analytical expression of $R_{0}$ in (9) is similar to the one in [29]. Moreover, the basic reproductive number for CCHF increased by a factor mentioned in (9) through co-feeding. The threshold quantities are comparable to [29]. In addition, the numerical approximation of $R_{0}$ and in [8] are comparable, and the magnitudes of the fitted parameters are in accord with previous findings [8,30]. Simulated CCHFV prevalence in the tick and the livestock populations resulting from our mechanistic model is of similar magnitude as noted in [19].

Sensitivity analysis reveals that the non-systemic transmission is not equally important as of the transmission cycle between the vertebrates and ticks and similar account has been noted in [69]. Some investigators have already performed a sensitivity analysis of $R_{0}$ [8,19,31], and we extend these findings, as we analyse the sensitivity on another important epidemiological quantity, i.e., the total number of infected humans. Similar to [19,31], our findings show that host density, duration of infection and the immune responses are sensitive parameters.

While CCHFV has been studied in [29] with livestock as the primary host, the systematic exploration of the model parameters and the mathematical illustrations of different control measures have not yet been fully explored. For tick-borne diseases, the use of acaricides is an established treatment, which primarily targets the tick population, but in some poor regions, this may not be feasible. However, one should bear in mind that the profuse usage of acaricides [29] may have a detrimental effect on the environment and can lead to resistant tick populations. Alternatives to using acaricides have been proposed, e.g., keeping chickens together with other livestock, because chickens eat ticks and may therefore reduce the risk of exposure to CCHFV [70].

Our modelling effort is novel in the sense that it aptly catches the potential routes of CCHF transmission burden for different countries and the approach to control it accordingly. We also methodically explore all possible ways to curb CCHF cases in humans. We find that the parameters of CCHFV dissemination vary in different endemic countries. Our results show that human sanitation and isolation are effective ways to reduce the CCHF cases in humans along with acaricide treatment of livestock as mentioned in [71] in economically deprived countries.

Just like other modelling endeavours on CCHF, our model has several limitations [29,30]. The model contains simplifications while pertaining to the feasible routes of CCHFV transmission. It is based on assumptions, where knowledge and parameters are missing. Variables are parametrised with values from the literature, which may be accurate or not, generally applicable or not. Therefore, the simulations conducted with our model are only meant for demonstration purposes. We recommend to parametrise the model for the specific situation, if it is used to plan or evaluate control measures.

Future studies with this model should include the proper investigation of the data related to CCHF and systematic explorations of the parameter space to find the necessary paths to reduce the disease prevalence effectively. Other investigators observed seasonality in human incidence and an effect of ambient temperatures [72]. These factors are not included in our approach. In addition, in urban areas of Pakistan, the risk of transmission is higher during the time of Eid-ul-Azha, when Muslims slaughter livestock animals [15]. The movement of animals and the migration of humans are also not included in this study, although they may be important variables.

Despite the limitations of our model, the analytical expression of $R_{0}$ and the mathematically exploration of control strategies may make it relevant in the fields of epidemiology and public health. Our work highlights the potential causes of CCHF outbreaks. The insights derived can pioneer the development of data-driven control measures modelling with scenarios and parameter values that are more realistic and adapted to a specific country or region. We expect that our work on CCHF spread and control measures may help to collect the necessary data related to CCHF and to further developing this and similar mathematical models and analyses.

## Figures and Tables

**Table 1:** Parameters used in the model (1)–(4), (6).

Parameter	Description	Value or Range	Units	References
$\pi_{L}$	Birth rate of livestock population	$\left[0.5,1.5\right]$	Livestock/Time	[39]
$\pi_{H}$	Birth rate of human population	$\left[0.5,1.5\right]$	Human/Time	[39]
$\pi_{T}$	Birth rate of tick population	$\left[0.5,3.5\right]$	Tick/Time	[40]
$\mu_{L}$	Death term of livestock population	$\left[1/3600,1/360\right]$	1/Time	[39]
$\mu_{H}$	Death term of human population	$\left[1/365\times 60,1/365\times 40\right]$	1/Time	[39]
$1/e_{T}$	Incubation period in tick	$\left[1,3\right]$	Time	[8,41]
$1/e_{L}$	Incubation period in livestock	$\left[3,5\right]$	Time	[8]
$1/e_{H}$	Incubation period in human	$\left[1,9\right]$	Time	[41]
ε	Proportion of hatched infected ticks	$\left[0.14,0.2\right]$	Number	[43]
$1/\alpha_{L}$	Recovery period of livestock	$\left[14,21\right]$	Time	[19]
$1/\alpha_{H}$	Recovery period of the human population	$\left[15,21\right]$	Time	[41,42]
$\sigma_{1}$	Transmission parameter: livestock to tick	$\left[0.11,0.33\right]$	Number	[43]
$\sigma_{2}$	Transmission parameter: tick to tick	$\left[0.01,0.04\right]$	Number	[8]
$\sigma_{3}$	Transmission parameter: tick to livestock	$\left[0.13,0.71\right]$	Number	[43]
$\sigma_{4}$	Transmission parameter: tick to human	$\left[0.25,0.375\right]$	Number	[43]
$\sigma_{5}$	Transmission parameter: livestock to human	$\left[0.001,0.002\right]$	Number	[39]
$\sigma_{6}$	Transmission parameter: human to human	$\left[0.26,0.75\right]$	Number	[46]
$\delta_{H}$	CCHF-induced death	$\left[0.3,0.8\right]$	1/Time	[18,41]
$\omega_{T}$	Mean number of eggs	$\left[4258,9476\right]$	Number	[8]
$\nu_{T}$	Strength of density-dependence in birth rate	0.025	1/Tick Time	[34,35]
$\sigma_{T}$	Detachment rate of tick	0.256	1/Time	[8]
$\omega_{1}$	Contribution of the rodent population	0.4	Number	[35]
$\omega_{2}$	Contribution of the livestock population	0.04	Number	[35]
$p_{T}$	Transmission efficiency: livestock to tick	$\left[0.11,0.33\right]$	Number	[8]
$\gamma_{T}$	Duration of the infective period	$\left[2,6\right]$	Time	[8]
$d_{T}$	Duration of attachment	$\left[6,8\right]$	Time	[8]
$N_{T}$	Rate of the average number of ticks feeding on livestock	$\left[5.5,8.5\right]$	Ticks/Time	[8]

## Data Availability

We have collected the publicly available data from [11,15,65,66,67,68].

## Supplementary Materials

Supplementary Materials: The following supporting information can be downloaded at: https://www.mdpi.com/article/10.3390/epidemiologia3010010/s1 refs. [8,15,19,31,34,35,39,44,57,73,74,75] are cited in the supplementary materials.
