# Supplementary material for: Ticks on the Run: A Mathematical Model of Crimean-Congo Haemorrhagic Fever (CCHF)—Key Factors for Transmission
Source: Epidemiologia (Basel). 2022 Mar 11;3(1):116–34. doi: 10.3390/epidemiologia3010010 (PMC9620928; doi:10.3390/epidemiologia3010010)
Supplement: Supplementary file 1 [file epidemiologia-03-00010-s001.zip › epidemiologia-1434433-supplementary.pdf]

Supplementary Information: Ticks on the Run: A Mathematical Model of Crimean-Congo Haemorrhagic Fever (CCHF)—Key Factors for Transmission

Suman Bhowmick, Khushal Khan Kasi, Jörn Gethmann, Susanne Fischer, Franz J. Conraths, Igor M. Sokolov, Hartmut H. K. Lentz

Contents

S1 Behaviour of solution **3**

S2 Model Parameters **4**

S3 Basic reproduction number **5**
- S3.1 Tick-Human-Livestock Model . . . 5
- S3.2 Tick-Human-human-Livestock Model . . . 5
- S3.3 Tick-Human Model . . . 6

S4 Mathematical properties of DFE **6**
- S4.1 Jacobian stability analysis . . . 6

S5 Tick Parameters and transmission parameters **9**

S6 Expression of R_T **9**

S7 Impact of seasonally varying rodent density **10**

S8 Impact of contact rates **11**

S9 Human-to-human transmission term **11**

S10 Initial condition and variables **12**

S11 Control strategies in different geographic locations **13**

S12 Summary of sensitivity analysis **14**

S13 Initial conditions for estimation **14**

S13.1 Fitted transmission parameters 15

S13.2 Parameter estimation statistics 16

S1 Behaviour of solution

Theorem S1 *All the solution trajectories of the model system (mentioned in the manuscript) initiating inside* Γ*, will remain within the interior of* Γ.

$$\Gamma = \Gamma_T \times \Gamma_L \times \Gamma_H$$

$$\Gamma_T = \left\{ (T_S, T_E, T_I) : 0 \leq T_S, T_E, T_I \leq \frac{\pi_T}{\mu_T} \right\}$$

$$\Gamma_L = \left\{ (L_S, L_E, L_I, L_R) : 0 \leq L_S, L_E, L_I, L_R \leq \frac{\pi_L}{\mu_L} \right\}$$

$$\Gamma_H = \left\{ (H_S, H_E, H_I, H_R) : 0 \leq H_S, H_E, H_I, H_R \leq \frac{\pi_H}{\mu_H} \right\}$$

Theorem S2 *The solution of the model system is positive,* $\forall t \geq 0$.

S2 Model Parameters

Parameter	Value	Reference
n_{Ro}	0.013	Bolzoni *et al.* (2012)
l_{Ro}	0.013	Bolzoni *et al.* (2012)
θ	0.55	Bolzoni *et al.* (2012)
σ_{Ro}	0.003676	Bolzoni *et al.* (2012)
σ_L	0.127	Bolzoni *et al.* (2012)
f_S	0.1	Matser *et al.* (2009)
d_{feed_L}	5	Hoch *et al.* (2018)
d_{feed_N}	8	Hoch *et al.* (2018)
d_{feed_A}	14	Hoch *et al.* (2018)
p_{T_i}	0.45-0.8	Hoch *et al.* (2018)
N_{Ro}	2.5-8.8	Hoch *et al.* (2018)
L_{Ro}	2.5-8.8	Hoch *et al.* (2018)
ε	0-0.2	Rosà and Pugliese (2007)
A_L	10	Bolzoni *et al.* (2012)
β_H	$[0.5, 0.75]$	Mondal *et al.* (2017)
η_H	$[0.0005, 0.0075]$	Mondal *et al.* (2017)
λ_H	0.85	Mondal *et al.* (2017)
τ_H	0.65	Mondal *et al.* (2017)
γ_H	$[0, 0.075]$	Mondal *et al.* (2017)
σ_H	0.90	Mondal *et al.* (2017)
μ_0	0.28	Hoch *et al.* (2018)
α_0	0.1	Hoch *et al.* (2018)

Table S1: Model Parameters

S3 Basic reproduction number

S3.1 Tick-Human-Livestock Model

$$
\mathcal{F}_{\mathcal{TL}} = \begin{pmatrix} 0 & \frac{T_S^* \sigma_2}{T} & 0 & \frac{T_S^* \sigma_1}{L} & 0 & 0 \\ 0 & 0 & 0 & 0 & 0 & 0 \\ 0 & \frac{L_S^* \sigma_3}{L} & 0 & 0 & 0 & 0 \\ 0 & 0 & 0 & 0 & 0 & 0 \\ 0 & \frac{H_S^* \sigma_4}{H} & 0 & \frac{H_S^* \sigma_5}{H} & 0 & 0 \\ 0 & 0 & 0 & 0 & 0 & 0 \end{pmatrix}
$$

and

$$
\mathcal{V}_{\mathcal{TL}} = \begin{pmatrix} e_T + \mu_T & 0 & 0 & 0 & 0 & 0 \\ -e_T & \mu_T - \varepsilon \pi_T & 0 & 0 & 0 & 0 \\ 0 & 0 & e_L + \mu_L & 0 & 0 & 0 \\ 0 & 0 & -e_L & \alpha_L + \mu_L & 0 & 0 \\ 0 & 0 & 0 & 0 & e_H + \mu_H & 0 \\ 0 & 0 & 0 & 0 & -e_H & \alpha_H + \delta_H + \mu_H \end{pmatrix}.
$$

To find the next generation matrix $\mathcal{K}_{\mathcal{TL}}^{\mathcal{L}} = -\mathcal{F}_{\mathcal{TL}} \mathcal{V}_{\mathcal{TL}}{}^{-1}$, we can further reduce $\mathcal{K}_{\mathcal{TL}}^{\mathcal{L}}$ to $\mathcal{K}_{\mathcal{TL}}$ according to Diekmann *et al.* (2010).

S3.2 Tick-Human-human-Livestock Model

$$
\mathcal{F}_{\mathcal{TLH}} = \begin{pmatrix} 0 & \frac{T_S^* \sigma_2}{T} & 0 & \frac{T_S^* \sigma_1}{L} & 0 & 0 \\ 0 & 0 & 0 & 0 & 0 & 0 \\ 0 & \frac{L_S^* \sigma_3}{T} & 0 & 0 & 0 & 0 \\ 0 & 0 & 0 & 0 & 0 & 0 \\ 0 & \frac{H_S^* \sigma_4}{T} & 0 & \frac{H_S^* \sigma_5}{L} & 0 & \frac{H_S^* \sigma_6}{H} \\ 0 & 0 & 0 & 0 & 0 & 0 \end{pmatrix}
$$

$$
\mathcal{V}_{\mathcal{TLH}} = \begin{pmatrix} e_T + \mu_T & 0 & 0 & 0 & 0 & 0 \\ -e_T & -\Pi_T \epsilon + \mu_T & 0 & 0 & 0 & 0 \\ 0 & 0 & e_L + \mu_L & 0 & 0 & 0 \\ 0 & 0 & -e_L & \alpha_L + \mu_L & 0 & 0 \\ 0 & 0 & 0 & 0 & e_H + \mu_H & 0 \\ 0 & 0 & 0 & 0 & -e_H & \alpha_H + \delta_H + \mu_H \end{pmatrix}
$$

To find the next generation matrix $\mathcal{K}^{\mathcal{L}}_{\mathcal{TLH}} = -\mathcal{F}_{\mathcal{TLH}}\mathcal{V}_{\mathcal{TLH}}{}^{-1}$, we can further reduce $\mathcal{K}^{\mathcal{L}}_{\mathcal{TLH}}$ to $\mathcal{K}_{\mathcal{TLH}}$ according to Diekmann *et al.* (2010).

S3.3 Tick-Human Model

$$\mathcal{F}_{\mathcal{TH}} = \begin{pmatrix} 0 & \frac{T_S^*\sigma_2}{T} & 0 & 0 \\ 0 & 0 & 0 & 0 \\ 0 & \frac{H_S^*\sigma_4}{T} & 0 & \frac{H_S^*\sigma_6}{H} \\ 0 & 0 & 0 & 0 \end{pmatrix}$$

$$\mathcal{V}_{\mathcal{TH}} = \begin{pmatrix} e_T + \mu_T & 0 & 0 & 0 \\ -e_T & -\pi_T\epsilon + \mu_T & 0 & 0 \\ 0 & 0 & e_H + \mu_H & 0 \\ 0 & 0 & -e_H & \alpha_H + \delta_H + \mu_H \end{pmatrix}.$$

To find the next generation matrix $\mathcal{K}^{\mathcal{L}}_{\mathcal{TH}} = -\mathcal{F}_{\mathcal{TH}}\mathcal{V}_{\mathcal{TH}}{}^{-1}$, we can further reduce $\mathcal{K}^{\mathcal{L}}_{\mathcal{TH}}$ to $\mathcal{K}_{\mathcal{TH}}$ according to Diekmann *et al.* (2010).

S4 Mathematical properties of DFE

S4.1 Jacobian stability analysis

To perform the stability analysis, we discard the expressions containing R in the model system because its value can be determined if we know the values of S_i, E_i, I_i, where $i = T, L, H$ respectively. Therefore, we have the following reduced system,

$$
\begin{aligned}
\frac{dT_S}{dt} &= \pi_T - \frac{\sigma_1 T_S L_I}{L} - \frac{\sigma_2 T_S T_I}{T} - \mu_T T_S + (1-\varepsilon)\pi_T T_I \qquad \text{(S1)}\\
\frac{dT_E}{dt} &= \frac{\sigma_1 T_S L_I}{L} + \frac{\sigma_2 T_S T_I}{T} - \mu_T T_E - e_T T_E\\
\frac{dT_I}{dt} &= e_T T_E - \mu_T T_I + \varepsilon \pi_T T_I\\
\frac{dL_S}{dt} &= \pi_L - \frac{\sigma_3 L_S T_I}{T} - \mu_L L_S\\
\frac{dL_E}{dt} &= \frac{\sigma_3 L_S T_I}{T} - e_L L_E - \mu_L L_E\\
\frac{dL_I}{dt} &= e_L L_E - \alpha_L L_I - \mu_L L_I\\
\frac{dH_S}{dt} &= \pi_H - \frac{\sigma_4 H_S T_I}{T} - \frac{\sigma_5 H_S L_I}{L} - \mu_H H_S\\
\frac{dH_E}{dt} &= \frac{\sigma_4 H_S T_I}{H} + \frac{\sigma_5 H_S L_I}{H} - e_H H_E - \mu_H H_E\\
\frac{dH_I}{dt} &= e_H H_E - \alpha_H H_I - \mu_H H_I - \delta_H H_I
\end{aligned}
$$

To analyse the local stability at E_0, we found the Jacobian of the reduced system (S1)

$$
\mathcal{J} = \begin{pmatrix}
-a_{11} & 0 & a_{13} & 0 & 0 & -a_{16} & 0 & 0 & 0\\
a_{21} & -a_{22} & a_{23} & 0 & 0 & a_{26} & 0 & 0 & 0\\
0 & a_{32} & -a_{33} & 0 & 0 & 0 & 0 & 0 & 0\\
0 & 0 & -a_{43} & -a_{44} & 0 & 0 & 0 & 0 & 0\\
0 & 0 & a_{53} & a_{54} & -a_{55} & 0 & 0 & 0 & 0\\
0 & 0 & 0 & 0 & a_{65} & -a_{66} & 0 & 0 & 0\\
0 & 0 & -a_{73} & 0 & 0 & -a_{76} & -a_{77} & 0 & 0\\
0 & 0 & a_{83} & 0 & 0 & a_{86} & a_{87} & -a_{88} & 0\\
0 & 0 & 0 & 0 & 0 & 0 & 0 & a_{98} & -a_{99}
\end{pmatrix}
$$

where,

$$
\begin{aligned}
a_{11} &= 1 - \varepsilon - \mu_T - \frac{L_I\sigma_1}{L} - \frac{T_I\sigma_2}{T} & a_{13} &= \frac{T_S\sigma_2}{T} & a_{16} &= \frac{T_S\sigma_1}{L} \\
a_{21} &= \frac{L_I\sigma_1}{L} + \frac{T_I\sigma_2}{T} & a_{22} &= e_T + \mu_T & a_{23} &= \frac{T_S\sigma_2}{T} & a_{26} &= \frac{T_S\sigma_1}{L} \\
a_{32} &= e_T & a_{33} &= \varepsilon\pi_T - \mu_T \\
a_{43} &= \frac{L_S\sigma_3}{L} & a_{44} &= \mu_T + \frac{T_I\sigma_3}{L} \\
a_{53} &= \frac{L_S\sigma_3}{L} & a_{54} &= \frac{T_I\sigma_3}{L} & a_{55} &= e_L + \mu_L \\
a_{65} &= e_L & a_{66} &= \alpha_L + \mu_L \\
a_{73} &= \frac{H_S\sigma_4}{L} & a_{76} &= \frac{H_S\sigma_5}{H} & a_{77} &= \mu_H + \frac{T_I\sigma_4}{L} + \frac{L_I\sigma_5}{H} \\
a_{83} &= \frac{H_S\sigma_4}{L} & a_{86} &= \frac{H_S\sigma_5}{H} & a_{87} &= \frac{T_I\sigma_4}{L} + \frac{L_I\sigma_5}{H} & a_{88} &= e_H + \mu_H \\
a_{98} &= e_H & a_{99} &= \alpha_H + \delta_H + \mu_H
\end{aligned}
$$

At the DFE (E_0), the Jacobian $\mathcal{J}(E_0)$ is

$$
\mathcal{J}(E_0) = \begin{pmatrix}
-a_{11}^0 & 0 & a_{13}^0 & 0 & 0 & -a_{16}^0 & 0 & 0 & 0 \\
0 & -a_{22}^0 & a_{23}^0 & 0 & 0 & a_{26}^0 & 0 & 0 & 0 \\
0 & a_{32}^0 & -a_{33}^0 & 0 & 0 & 0 & 0 & 0 & 0 \\
0 & 0 & -a_{43}^0 & -a_{44}^0 & 0 & 0 & 0 & 0 & 0 \\
0 & 0 & a_{53}^0 & 0 & -a_{55}^0 & 0 & 0 & 0 & 0 \\
0 & 0 & 0 & 0 & a_{65}^0 & -a_{66}^0 & 0 & 0 & 0 \\
0 & 0 & -a_{73}^0 & 0 & 0 & -a_{76}^0 & -a_{77}^0 & 0 & 0 \\
0 & 0 & a_{83}^0 & 0 & 0 & a_{86}^0 & 0 & -a_{88}^0 & 0 \\
0 & 0 & 0 & 0 & 0 & 0 & 0 & a_{98}^0 & -a_{99}^0
\end{pmatrix}
$$

where,

$$
\begin{aligned}
a_{11}^0 &= 1-\varepsilon-\mu_T & a_{13}^0 &= \frac{\pi_T^0\sigma_2}{T} & a_{16}^0 &= \frac{\pi_T^0\sigma_1}{L} \\
a_{22}^0 &= e_T+\mu_T & a_{23}^0 &= \frac{\pi_T^0\sigma_2}{T} & a_{26}^0 &= \frac{\pi_T^0\sigma_1}{L} \\
a_{32}^0 &= e_T & a_{33}^0 &= \varepsilon\pi_T^0-\mu_T \\
a_{43}^0 &= \frac{L_S^*\sigma_3}{L} & a_{44}^0 &= \mu_T \\
a_{53}^0 &= \frac{L_S^*\sigma_3}{L} & a_{55}^0 &= e_L+\mu_L \\
a_{65}^0 &= e_L & a_{66}^0 &= \alpha_L+\mu_L \\
a_{73}^0 &= \frac{H_S^*\sigma_4}{L} & a_{76}^0 &= \frac{H_S^*\sigma_5}{H} & a_{77}^0 &= \mu_H \\
a_{83}^0 &= \frac{H_S^*\sigma_4}{L} & a_{86}^0 &= \frac{H_S^*\sigma_5}{H} & a_{88}^0 &= e_H+\mu_H \\
a_{98}^0 &= e_H & a_{99}^0 &= \alpha_H+\delta_H+\mu_H
\end{aligned}
$$

After finding the characteristic polynomial of $\mathcal{J}(E_0)$ and applying *Routh-Hurwitz* criterion, we can claim following Mpeshe *et al.* (2011) that the model system S.1 has a unique DFE(E_0) which is locally asymptotically stable.

S5 Tick Parameters and transmission parameters

According to Hoch *et al.* (2018)

$$\mu_T = \mu_0 + \alpha_0 \ln(1 + \frac{T_i}{L}) \tag{S2}$$

where i stands for S, E, I.

S6 Expression of R_T

If we exclude the CCHFV transmission through co-feeding, we have the basic reproduction number as follows:

$$R_{LA}^{\mathcal{W}} = \sqrt{R_{LA}} \text{ where } \mathcal{W} \text{ stands for without co-feeding} \tag{S3}$$

$$R_T = \left[R_{LA}\left(1 + \frac{R_{LA}^{\mathcal{W}}}{R_{LA}}\right)\left(1 - \frac{R_{LA}^{\mathcal{W}}}{R_{LA}}\right)\right] \tag{S4}$$

S7 Impact of seasonally varying rodent density

Seasonal fluctuations in rodent density have a positive influence in driving the variation in the prevalence of CCHF in the ticks. Additionally, we set up a simulation to understand the influence of seasonally varying density of the rodents on the prevalence of CCHF in the ticks. We therefore modified the rodents density $\varrho(t) \mapsto \varrho^{In} + \delta F(t)$, where ϱ^{In} is the initial density, δ is the strength of the changes, i.e. positive δ leads to an increasing ϱ with time, while negative a leads to a decreasing ϱ with time and $F(t)$ is a generic trigonometric function. We explored the influence of seasonally varying the density of the rodents on the prevalence of CCHF in the ticks. The equation of the birth term of the ticks is as follows: $\pi_T = \sigma_T \omega_T \exp\left(-\gamma_T \frac{T_S+T_E+T_I}{\varrho(t)\omega_1+L\omega_2}\right)$. To incorporate the seasonality in the easiest way, we set $\varrho(t) = \varrho_1 + \varrho\cos(\delta t)$. Due to the lack of information about the seasonally varying density of the rodents, we have chosen $\delta = 1$, then after running the simulation, we have the following, when ϱ_1 is 100 and ϱ is as mentioned in the main-text. Figure S1 reveals that the impact of seasonally varying carrying capacity of Rodent (ϱ)

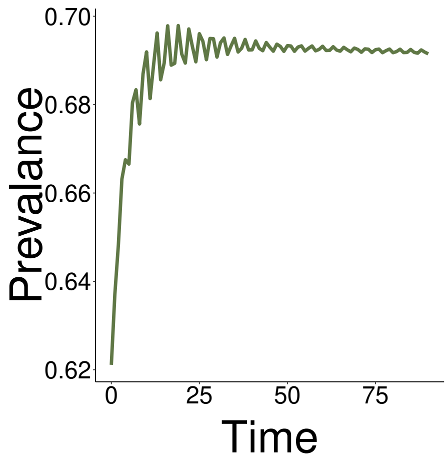

Figure S1: Relationship between seasonally varying density of rodents and the CCHFV prevalence in the adult ticks. The values of the parameters are taken from the Table 2 in the main text.

S8 Impact of contact rates

With the purpose to understand the influence of the contact rates, we introduce a scaling factor η on the host-specific infection rates σ_1 and σ_3 following Nguyen *et al.* (2019). After using the scaling factor η, the host-specific infection rates change to $\sigma_1 \to \eta\sigma_1$ and $\sigma_3 \to \eta\sigma_3$. Now, we compute the values of basic reproduction number (R_{LA}) for the values of $\eta \in [0, 1]$. Figure S2 represents the graphical representation of the relationship between η and R_{LA}.

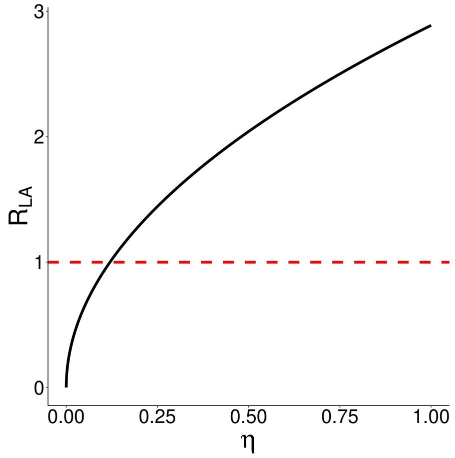

Figure S2: Relationship between basic reproduction number (R_{LA}) and the scaling factor (η). The values of the parameters are taken from the Table 2 in the manuscript.

Figure S2 reveals that the value of R_{LA} is less than unity, when $\eta \approx 0.18$. This simulation suggests that curbing the contact of adult ticks and livestock to less than 18% may help to reduce the number of CCHF cases.

S9 Human-to-human transmission term

According to Mondal *et al.* (2017), the human to transmission has been modelled as

$$\sigma_6 = \beta_H(1 - \eta_H\lambda_H\tau_H)(1 - \gamma_H\lambda_H\sigma_H) \qquad \text{(S5)}$$

S10 Initial condition and variables

Variable	Description of Model Variables
T_S	Susceptible ticks
T_E	Exposed ticks
T_I	Infected ticks
L_S	Susceptible livestock
L_E	Exposed livestock
L_I	Infected livestock
L_R	Recovered livestock
H_S	Susceptible humans
H_E	Exposed humans
H_I	Infected humans
H_R	Recovered humans
T	Total tick population
L	Total livestock population
ϱ	Total rodent population
H	Total human population

Table S2: Variables used in the model as described in the main text.

Variable	Initial Value
T_S	30
T_E	10
T_I	10
L_S	970
L_E	20
L_I	10
L_R	0
H_S	99
H_E	0
H_I	1
H_R	0

Table S3: Initial values for the simulations.

S11 Control strategies in different geographic locations

Combined Control If we combine both the control options then our target set is $\mathcal{S} = \{(1,2),(2,1),(3,1),(3,2),(3,3)\}$. Target reproduction number $\mathcal{T}_{\mathcal{S}}$ with respect to $\mathcal{S}$ is

$$\boldsymbol{\rho}\begin{pmatrix} 0 & K_{12} & 0 \\ \frac{K_{21}}{1-K_{11}} & 0 & 0 \\ \frac{K_{31}}{1-K_{11}} & K_{32} & K_{33} \end{pmatrix} = \max\left\{K_{33}, \sqrt{\frac{K_{12}K_{21}}{1-K_{11}}}\right\} \tag{S6}$$

Isolation: It is difficult to prevent or control the CCHFV infection cycle in livestock and ticks, as the tick–animal–tick cycle usually goes unnoticed, and CCHFV infection in livestock is not evident due to the lack of clinical symptoms in animals. Moreover, the abundance of tick vectors is widespread and large in numbers, which requires an efficient tick control strategy. This may be possible mainly in structured livestock farms. In farms, where tick control may not be possible due to economic or other constraints Atif *et al.* (2017); only isolation could be a realistic option. In this situation the target set is $\mathcal{S} = \{(3,3)\}$.

$$\boldsymbol{\rho}\begin{pmatrix} 0 & 0 & 0 \\ 0 & 0 & 0 \\ K_{33}\left(\frac{K_{21}\left(\frac{K_{12}K_{31}}{K_{11}-1}-K_{32}\right)}{(K_{11}-1)\left(\frac{K_{12}K_{21}}{k_{11}-1}+1\right)} - \frac{k_{31}}{k_{11}-1}\right) & -\frac{\left(\frac{K_{12}K_{31}}{K_{11}-1}-K_{32}\right)K_{33}}{\frac{K_{12}K_{21}}{K_{11}-1}+1} & K_{33} \end{pmatrix} = K_{33} \tag{S7}$$

It is interesting to note from the mathematical perspective that the efforts required to eradicate the disease are same for both *Human Sanitation & Isolation* and only for *Isolation.* This can be attributed to the fact that the latter case is a subset of the former control method.

S12 Summary of sensitivity analysis

	original	bias	std. error	min. c.i.	max. c.i.
π_T	0.37	0.001	0.04	0.25	0.50
σ_1	0.40	-0.004	0.04	0.28	0.53
σ_2	0.40	0.002	0.04	0.28	0.54
μ_T	-0.89	0.002	0.01	-0.92	-0.86
e_T	0.35	-0.009	0.04	0.24	0.51
π_L	0.23	-0.008	0.05	0.08	0.38
σ_3	0.52	0.005	0.04	0.42	0.63
μ_L	-0.22	0.001	0.05	-0.38	-0.08
e_L	0.27	-0.009	0.05	0.13	0.45
α_L	0.09	-0.003	0.05	-0.05	0.23
π_H	0.20	0.002	0.04	0.06	0.34
σ_4	0.29	0.004	0.05	0.14	0.45
σ_5	0.18	-0.003	0.05	0.04	0.34
μ_H	0.19	0.002	0.05	0.05	0.37
e_H	0.22	0.003	0.05	0.08	0.38
α_H	0.22	-0.009	0.05	0.06	0.38
δ_H	0.26	-0.006	0.05	0.12	0.41

S13 Initial conditions for estimation

Different initial conditions (IC) are used for the data fitting. Here, first we list the ICs for the human population of the concerned countries. For Iran it is (487, 2, 1, 2900, 29, 20, 23), Afghanistan it is (370, 3, 8, 1208, 29, 38, 4), Bulgaria it is (241, 23, 4, 1278, 3, 3, 2), Turkey it is (569, 23, 29, 1589, 68, 198, 8), Pakistan it is (444, 25, 3, 897, 30, 35, 6), and for Kosovo it is (200, 9, 2, 238, 23, 9, 2). We have used Hegde (2019) for the ICs.

S13.1 Fitted transmission parameters

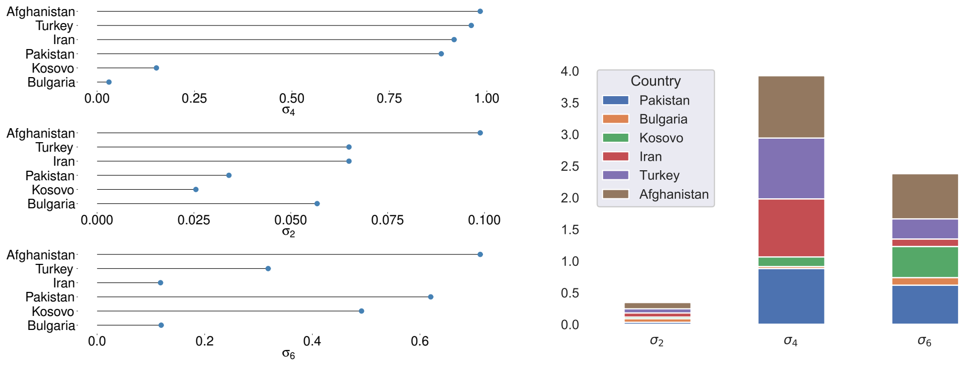

(a) (b)

Figure S3: Differences among the parameter sets of the considered countries. (a) List of fitted transmission parameters (b) Stack-bar plot of the fitted transmission parameters of different countries from the model fitting.

Parameter	Pakistan	Bulgaria	Kosovo	Iran	Turkey	Afghanistan
σ_2	0.03403	0.0568	0.0255	0.06502	0.06502	0.0989
σ_4	0.883	0.03046	0.15219	0.9160	0.960	0.983
σ_6	0.6200	0.1191	0.4914	0.1179	0.3179	0.712

The values of the fixed parameters such as π_T, π_H etc are mentioned in the main text.

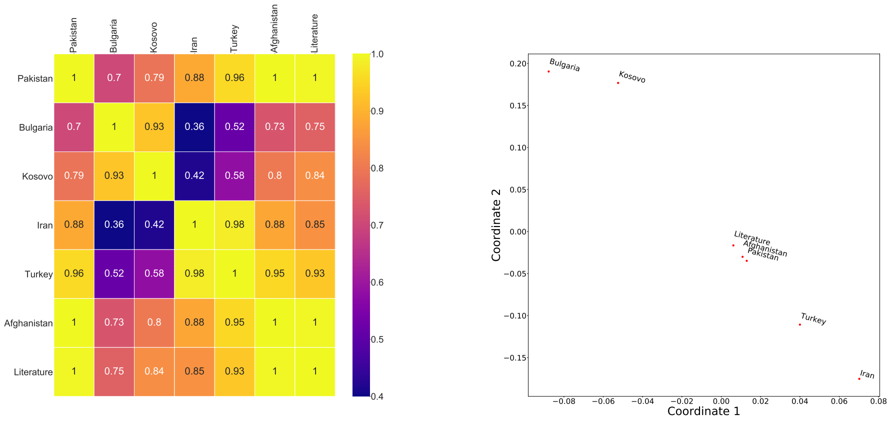

(a) (b)

Figure S4: Differences in the parameter sets of the considered countries. (a) Cosine similarity matrix of the fitted transmission parameters and the values from the literatures Mondal *et al.* (2017); Bolzoni *et al.* (2012); Rosà and Pugliese (2007); Hoch *et al.* (2016) (b) Generalised spatial embedding of the cosine distances amongst the estimated infection parameters and literature values.

S13.2 Parameter estimation statistics

Calculated Parameter Values

Country	Parameter	Initial guess	Final estimate	Standard error	t	Prob(t)
Pakistan	σ_2	0.0056	0.03403	16.5969	1.51	0.06502
	σ_4	0.00288	0.883	14.9690	2.18	0.960
	σ_6	0.0007	0.6200	11.9148	7.01	0.3179

Country	Parameter	Initial guess	Final estimate	Standard error	t	Prob(t)
Bulgaria	σ_2	0.0036	0.0568	1.5969	1.51	0.0542
	σ_4	0.0068	0.03046	4.9690	2.18	0.1867
	σ_6	0.0001	0.1191	11.9148	4.18	0.1999

Country	Parameter	Initial guess	Final estimate	Standard error	t	Prob(t)
	σ_2	0.0021	0.0255	1.5969	0.51	0.472
Kosovo	σ_4	0.0095	0.15219	4.9690	0.18	0.867
	σ_6	0.0001	0.4914	11.9148	0.88	0.32

Country	Parameter	Initial guess	Final estimate	Standard error	t	Prob(t)
	σ_2	0.0047	0.06502	1.5969	7.91	0.472
Iran	σ_4	0.0055	0.9160	15.9942	5.18	0.0571
	σ_6	0.0041	0.1179	1.9918	6.88	0.0826

Country	Parameter	Initial guess	Final estimate	Standard error	t	Prob(t)
	σ_2	0.0061	0.06502	0.8591	17.18	0.672
Turkey	σ_4	0.0063	0.960	15.9942	1.897	0.5871
	σ_6	0.0076	0.3179	1.9918	6.014	0.526

Country	Parameter	Initial guess	Final estimate	Standard error	t	Prob(t)
	σ_2	0.0061	0.0989	0.8591	47.98	0.0907
Afghanistan	σ_4	0.0063	0.9830	45.0993	16.805	0.01819
	σ_6	0.0076	0.7120	31.0907	12.784	0.0781

References

Hoch, T.; Breton, E.; Josse, M.; Deniz, A.; Guven, E.; Vatansever, Z. Identifying main drivers and testing control strategies for CCHFV spread. *Exp. Appl. Acarol.* **2016**, *68*, 347–359.

Hoch, T.; Breton, E.; Vatansever, Z. Dynamic Modeling of Crimean Congo Hemorrhagic Fever Virus (CCHFV) Spread to Test Control Strategies. *J. Med Entomol.* **2018**, *55*, 1124–1132.

Matser, A.; Hartemink, N.; Heesterbeek, H.; Galvani, A.; Davis, S. Elasticity analysis in epidemiology: An application to tick-borne infections. *Ecol. Lett.* **2009**, *12*, 1298–1305.

Mondal, M.K.; Hanif, M.; Biswas, M.H.A. A mathematical analysis for

controlling the spread of Nipah virus infection. *Int. J. Model. Simul.* **2017**, *37*, 185–197.

Nguyen, A.; Mahaffy, J.; Vaidya, N.K. Modeling transmission dynamics of lyme disease: Multiple vectors, seasonality, and vector mobility. *Infect. Dis. Model.* **2019**, *4*, 28–43.

Rosà, R.; Pugliese, A. Effects of tick population dynamics and host densities on the persistence of tick-borne infections. *Math. Biosci.* **2007**, *208*, 216–240.

Mpeshe, S.C.; Haario, H.; Tchuenche, J.M. A Mathematical Model of Rift Valley Fever with Human Host. *Acta Biotheor.* **2011**, *59*, 231–250.

Atif, M.; Saqib, A.; Ikram, R.; Sarwar, M.R.; Scahill, S. The reasons why Pakistan might be at high risk of Crimean Congo haemorrhagic fever epidemic; a scoping review of the literature. *Virol. J.* **2017**, *14*, 63.

Bolzoni, L.; Rosà, R.; Cagnacci, F.; Rizzoli, A. Effect of deer density on tick infestation of rodents and the hazard of tick-borne encephalitis. II: Population and infection models. *Int. J. Parasitol.* **2012**, *42*, 373–381.

Diekmann, O.; Heesterbeek, J. A. P.; Roberts, M. G. The construction of next-generation matrices for compartmental epidemic models. *J. R. Soc. Interface.* **2010**, *47*, 873-885.

Baghi, H. B.; Aghazadeh, M. Include Crimean-Congo haemorrhagic fever virus prevention in pre-travel advice. *Travel Med. Infect Dis.* **2016**, *14*, 634 - 635.

Hegde, N. Livestock Development for Sustainable Livelihood of Small Farmers. *Asian J. Anim. Vet. Adv.* **2019**, *3*, 1 - 17.
